# Nanomaterials and Autophagy: New Insights in Cancer Treatment

**DOI:** 10.3390/cancers5010296

**Published:** 2013-03-21

**Authors:** Elisa Panzarini, Valentina Inguscio, Bernardetta Anna Tenuzzo, Elisabetta Carata, Luciana Dini

**Affiliations:** Department of Biological and Environmental Science and Technology (Di.S.Te.B.A.), University of Salento, Lecce 73100, Italy; E-Mails: elisa.panzarini@unisalento.it (E.P.); valenting@hotmail.it (V.I.); bernardetta.tenuzzo@unisalento.it (B.A.T.); elisabetta.carata@unisalento.it (E.C.)

**Keywords:** nanomaterials, autophagy, cancer, cancer therapy, epigenetic factors

## Abstract

Autophagy represents a cell’s response to stress. It is an evolutionarily conserved process with diversified roles. Indeed, it controls intracellular homeostasis by degradation and/or recycling intracellular metabolic material, supplies energy, provides nutrients, eliminates cytotoxic materials and damaged proteins and organelles. Moreover, autophagy is involved in several diseases. Recent evidences support a relationship between several classes of nanomaterials and autophagy perturbation, both induction and blockade, in many biological models. In fact, the autophagic mechanism represents a common cellular response to nanomaterials. On the other hand, the dynamic nature of autophagy in cancer biology is an intriguing approach for cancer therapeutics, since during tumour development and therapy, autophagy has been reported to trigger both an early cell survival and a late cell death. The use of nanomaterials in cancer treatment to deliver chemotherapeutic drugs and target tumours is well known. Recently, autophagy modulation mediated by nanomaterials has become an appealing notion in nanomedicine therapeutics, since it can be exploited as adjuvant in chemotherapy or in the development of cancer vaccines or as a potential anti-cancer agent. Herein, we summarize the effects of nanomaterials on autophagic processes in cancer, also considering the therapeutic outcome of synergism between nanomaterials and autophagy to improve existing cancer therapies.

## 1. Introduction

Recently, the field of cancer therapies has been improved by many diverse scientific disciplines in order to better fight cancer diseases. A new very interesting area of research is “nanotechnology” that involves the creation, manipulation and application of structures in the nanometer-size range, has is revolutionizing cancer diagnosis and therapy [1]. The field mainly studied is the targeted delivery of drug molecules to diseased areas, due to the critical and pharmacokinetically peculiar environment existing in tumours. In fact, nanocarriers could offer many advantages over free drugs, such as protection from degradation, selective and improved absorption into a selected tissue, and control of the pharmacokinetic and drug tissue distribution profile. Interestingly, since nanomaterials (NMs) can interact with the autophagic pathway, and since autophagy is strongly implicated in the positive outcome of cancer therapies, an emerging field of tumour research is currently studying engineered nanomaterials as exploitable tools in cancer therapies. This review examines the role of NMs in autophagy with a focus on correlation among NMs, autophagy and cancer featuring the possible development of autophagy-modulating strategies against cancer.

## 2. Nanomedicine, Nanomaterials and Application in Cancer

Nanomedicine represents an innovative and multidisciplinary field that exploits nanotechnology to utilize in disease detection, diagnosis and treatment nanoscale (1–100 nm) constructs called NanoMaterials-NMs and defined as “materials with lengths ranging from 1 to 100 nanometers in two or three dimensions” [2]. Based on nanotechnology, nanocarriers have been synthesized from organic and inorganic materials in order to enhance the performance of medicines, reduce systemic side effects and enhance therapeutic efficiency. A drug may be adsorbed or attached to or encapsulated in the nanocarriers. The targeting of molecules or drugs can be passive or active; the first exploits the characteristic features of target tissue biology, whereas in active approaches, nanocarriers are conjugated with molecules able to bind overexpressed antigens or receptors present on the target cell surface. The molecules bound on nanocarriers can be proteins (mainly antibodies and their fragments), nucleic acids (aptamers), or other receptor ligands (peptides, vitamins, and carbohydrates). In addition, active targeting can be also achieved through manipulation of physical stimuli (e.g., temperature, pH, magnetism) [3]. In the targeted diseased tissue, the drug is released in a controlled manner through changes in the physiological environment, such as temperature, pH, osmolality, or via some enzymatic activity.

**Figure 1:**
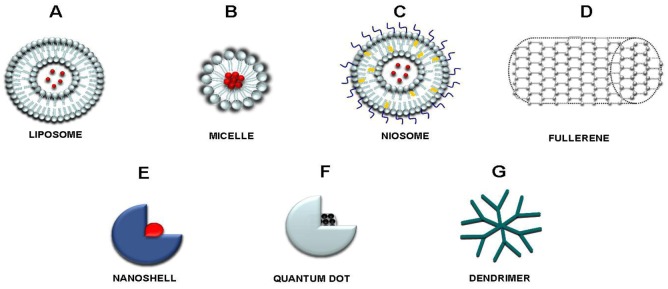
Drug delivery nanoparticle systems: (**A**) liposome; (**B**) micelle; (**C**) niosome; (**D**) fullerene; (**E**) nanoshell; (**F**) quantum dot; (**G**) dendrimer.

Nanocarriers exploited in medical applications: (a) are made from a biocompatible, well characterized, and easily functionalized material; (b) exhibit high differential uptake efficiency in ill cells compared to healthy cells; (c) are either soluble or colloidal in aqueous conditions to increase their effectiveness; (d) have an extended circulating half-life, a low rate of aggregation, and a long shelf life [4]. The main nanocarrier systems are liposomes, micelles, niosomes, nanoparticles, dendrimers and nanofibers. Liposomes (80–300 nm size range) were the first drug carriers investigated [5]. They are artificially prepared vesicles composed of monolamellar or multilamellar bilayers of phospholipids and steroids (e.g., cholesterol). They can transport both hydrophilic and hydrophobic molecules via encapsulation in their aqueous core or in their hydrophobic membrane respectively (Figure 1A) [6]. Micelles (10–100 nm size range) are spherical self-assemblies of amphiphilic-block copolymers in an aqueous environment, consisting of a poly(ethylene glycol) (PEG) hydrophilic corona and a hydrophobic core, composed of polymers like poly(ε-caprolactone) (PLC) and poly(D,L-lactic acid) (PLA) allowing solubilization of lipophilic drugs (Figure 1B) [7]. Niosomes, structurally similar to liposomes, are non-ionic surfactant vesicles having a multilamellar or unilamellar bilayer structure. Niosomes are formed by hydration of non-ionic surfactant dried films and can entrap both hydrophilic and lipophilic drugs in the aqueous layer and vesicular membrane, respectively (Figure 1C) [8]. Nanoparticles (NPs) are natural or synthetic-prepared particles of less than 100 nm diameter that can have different properties and different release characteristics by forming matrix-type or reservoir-type structures, named nanospheres or nanocapsules. The nanoparticles include: (1) carbon-based nanoparticles, including fullerenes (Figure 1D) and single (SWCNTs)- and multi(MWCNTs)-walled carbon nanotubes; (2) metal-based nanoparticles, such as gold colloids, nanoshells (Figure 1E), nanorods, and superparamagnetic iron oxide nanoparticles (spherical nanocrystals with Fe^2+^ and Fe^3+^ cores); and (3), semiconductor-based nanoparticles such as quantum dots (QDs colloidal fluorescent semiconductor nanocrystals) (Figure 1F) [9]. Dendrimers are artificial macromolecules with tree-like structures in which the atoms are arranged in many branches and subbranches radiating out from a central core. Their architecture offers unique advantages since they can transport molecules both in their internal cavities or attached to their branches; moreover the branches can be exploited to attach functional groups improving the precise targeting (Figure 1G) [10]. Nanofibers are ultrafine polymer fibers with diameters ranging from tens of nanometers to 1 micron obtained by electrospinning a polymer solution. As a fibrous scaffold, nanofibers are able to entrap drugs with a high loading capacity and high encapsulation efficiency because of their low weight and inherent high surface-to-volume ratio [11].

It is well established that advances in nanoscience and nanotechnology have improved cancer imaging and diagnostic conventional methods, as reviewed in [12].

Conceivably, the greatest impact of nanotechnology in the field of oncology is in cancer therapy and specifically in the realm of drug/gene delivery. In fact, advances in protein engineering and materials science bring new hope to cancer patients by contributing to novel nanoscale targeting approaches. The tumour area is a very particular microenvironment that limits the efficacy of conventional chemotherapeutic drugs and nanocarriers can overcome this problem (see [13]). 

In fact, nanocarriers: (i) can carry a complex and highly concentrated therapeutic “payload”, (ii) can be attached to multivalent targeting ligands improving both affinity and specificity for target cells, (iii) can accommodate multiple drug molecules that can be simultaneously and serially released allowing combinatorial cancer therapy, and (iv) can bypass Multiple Drug Resistance (MDR), a typical shortcoming of traditional chemotherapeutics [14]. The first generation of nanodrug delivery systems used in cancer therapy were liposomes; whereas the latest generation are polymeric NPs loaded with molecules that functionalize their surface to mask the nanocarriers’ surface to immune cells, thus preventing recognition and elimination. The use of nanocarriers in chemotherapy (conventional cancer treatment) is well established and many therapeutic nanocarriers have been approved by the U.S. F.D.A. for wider use. A list of the most recent proposed cancer chemicals vehicled by NMs is reported in Table 1.

**Table 1:** Examples of nanoparticles delivered anti-cancer drugs.

Delivered drugs	Nanomaterials	Reference
Camptotechin	Si-NPs	[15]
Kahalalide F	Au-NPs	[16]
Docetaxel	Zn-NPs	[17]
Gemcitabine	immunoliposomes	[18]
Paclitaxel	cationic liposomes	[19]
Paclitaxel	tetrahexyloxy-tetra-*p*aminocalix[4]arene (A4C6)	[20]
Doxorubicin	polymalic acid backbone	[21]
Doxorubucin	PEG liposomes	[22]
Doxorubucin	PEG-LPD(liposomes polycation DNA)	[23]
Myocet (doxorubicin)	non PEG liposomes	[23]
DaunoXome (daunorubicin)	unilamellar liposomes	[24]
Cisplatin	MWCNTs	[25]
Gemcitabine	SWCNTs	[26]
Doxorubicin	MWCNTs@poly(ethylene glycol-b-propilene sulphide)	[27]
Cisplatin-EGF	SWNTs	[28]
Gemcitabine	Fe_3_O_4_@poly(ethylene glycol)-NPs	[29]
Doxorubicin	Fe_3_O_4_@gelatin-NPs	[30]
5-Fluorouracil	Fe_3_O_4_@ethylcellulose-NPs	[31]
Daunorubicin	Fe_3_O_4_-NPs	[32]
Cisplatin	Fe_3_O_4_@poly ε-caprolactone-NPs	[33]
Paclitaxel	Fe_3_O_4_@poly[aniline-co-sodium *N*-(1-butyric acid)]-NPs	[34]
1,3-Bis(2-chloroethyl)-1-nitrosourea (BCNU)	Fe_3_O_4_@poly[aniline-co-*N*-(1-butyric acid) aniline]	[35]

NMs are currently under evaluation in clinical trials for their ability to synergize not only with chemotherapy but also with new non-conventional cancer therapy, such as PhotoDynamic Therapy (PDT) or internal radiotherapy or gene therapy.

PDT is a two-step process involving the irradiation of photosensitizer (PS)-loaded cancer cells [36]. Several NMs are currently used to deliver PSs [37] and it has been demonstrated that some can also display photosensitizer activity. For example, graphene quantum dots are indicated as excellent candidates for PDT in U251 human glioma cells [38] and Au-NPs conjugated with water soluble purpurin results in an increased anti-cancer efficacy in A549 lung cancer cells [39]. 

Among new cancer treatment approaches, internal radiotherapy by using NPs is very promising for the management of refractory tumors [40,41]. This new treatment, called nanovectorized radiotherapy, is based on the use of NPs as a reservoir for radionuclides, that are thus specifically directed to cancer cells. This novel radiotherapy ensures the simultaneous cell killing and immune response through the use of peculiar biomaterials and/or surface ligands. Indeed, several studies have demonstrated that nanoscale drug delivery systems could activate the host immune system. For example, poly(D,L-lactide-co-glycolide (PLGA) NPs are able to stimulate dendritic cells (DCs) [42] through activation of NLRP3 inflammasome; poly(γ-glutamic acid) NPs induce DC maturation through the NFkB activation and MAPK pathways and can be used as a vaccine adjuvant [43,44,45]. Recently Vanpouille *et al.* [46] demonstrated that ruthenium-188-loaded NPs result in tumor rejection in long-term survivor animals through a potent stimulation of a tumour-specific immune response, *i.e*., IFNγ production, recruitment of immune effector T cells within the tumor area and memory response.

Another promising field of application of nanocarriers’ potentiality is cancer gene therapy. Cancer gene therapy offers the possibility to fight the pathology at the root by upregulation or downregulation of target genes. The success of gene therapy depends on the efficient delivery of nucleic acids into the cells. The conventional vectors used include viruses, such as adenoviruses and retroviruses, that induce immune responses; moreover, due to the presence of nucleases in the bloodstream and to the immune system recognition of foreign nucleic acids, circulating DNA and RNA have very short half-lives [47]. Nanocarriers have been proposed as a tool to circumvent these limitations: cationic liposomes and cationic polymers bind nucleic acids and enter cells by endocitosis [48]. Recently, magnetic nanoparticle technology has offered the possibility to achieve selective and efficient delivery of therapeutic genes by using external magnetic fields, and also allows simultaneous monitoring of the *in vivo* delivery. Compared to conventional gene delivery strategies, this technique has been shown to significantly increase gene delivery to human xenograft tumors models, as well as various internal organs (e.g., liver, kidney) and the central nervous system [49]. 

### 2.1. Nanosafety

The study of potential risks associated with the manufacture, use and disposal of nanoscale materials and their mechanisms of toxicity is very important, primarily in the field of biomedicine. More and more studies are trying to define any possible toxicity of engineered NMs [50]. Although the mechanisms of their toxicity are still undefined, oxidative stress and inflammation are the most widely accepted NMs toxic effects [51]. The continuous increase in the number and uses of nanotechnological products has recently stimulated interest in examining their long-term impact on genetic and epigenetic processes. If several studies (recently reviewed by Becker *et al.*, [52]) report that NMs could be genotoxic and carcinogenic only very few are investigating NMs-induced epigenetic changes leading to abnormal apoptosis, enhanced oxidative stress and pro-inflammatory effects [53]. Meng [54] reports that multi-hydroxylated endohedral metallofullerenol NPs exert an epigenetic modulation eliciting an antineoplastic action in MCF-7 human breast cancer. Moreover, silicon dioxide NPs (SiO_2_-NPs) induce oxidative and genomic stress in human keratinocyte HaCaT cells that cause cytotoxicity and protein alteration [55,56]. These effects could be mediated by a global hypoacetylation that implies an epigenetic response. In fact, the levels of DNA methylation and the related methyl transferase, DNMT1, DNMT3a and MDB2) decrease in a dose dependent manner at the mRNA and protein level [57].

In searching for new emerging mechanisms of action of NM toxicity, recent literature data strongly highlight the roles of autophagy and lysosomal dysfunction [58]. Most likely, autophagy induction may be used to degrade NMs, perceived by the cell as foreign body, via sequestration into autophagosomes [59]. Several papers suggest that this interaction could be exploited in cancer management.

The catabolic process of autophagy occurring under certain stress conditions, *i.e*., growth factors or nutrients or energy level depletion, and consists in the lysosomal degradation and/or recycling of the cytoplasmic material, such as cytosolic proteins, macromolecules, organelles, and protein aggregates. This process regulates cellular homeostasis and produces defective organelles, misfolded or aggregated proteins, and long-lived molecules turnover [60]. Three types of autophagy, *i.e*., macroautophagy [61], microautophagy [62], and chaperone-mediated autophagy [63], have been identified, whose differences depend on cargo delivery to lysosomes. In particular, in macroautophagy (hereafter referred to as autophagy), newly formed vesicles (named autophagosomes) sequester the material and deliver it to lysosomes for degradation [61]. Among the degradation products released into the cytoplasm, amino acids support new protein synthesis, while carbohydrates and lipids provide energy for cells [64]. The autophagic process, regulated by AuTophaGy-related genes (ATGs) originally identified in the yeast [65], consists of five phases: initiation (or nucleation), elongation, closure, maturation and degradation, whose morphological features have been widely characterized [66] and, recently, new guidelines for autophagy assessment have been indicated [67]. 

The levels of autophagy, in terms of induction or inhibition, are regulated via multiple sensors, such as mTOR, Beclin-1, p53 and Ras, and pathways, like class I PI3K, class III PI3K, LKB1/AMPK and ER stress response [68]. Recently, certain microRNA (miRNAs), a class of small non-coding RNAs that exert catalytic, structural or regulatory activities by annealing to specific target RNAs, and by downregulating their stability and/or translation [69], have emerged as important epigenetic modulators of the different autophagic stages [70].

Autophagy is involved in different diseases, such as infections, neurodegeneration, aging, Crohn’s disease, heart disease, and cancer [71]. In particular, excessive or prolonged autophagy as well deficient autophagy affect oncogenesis [72] and, consequently, cancer therapy [73].

## 3. Autophagy-Assisted Cancer Therapy: Old and New Insights

### 3.1. Autophagy and Cancer

**Figure 2:**
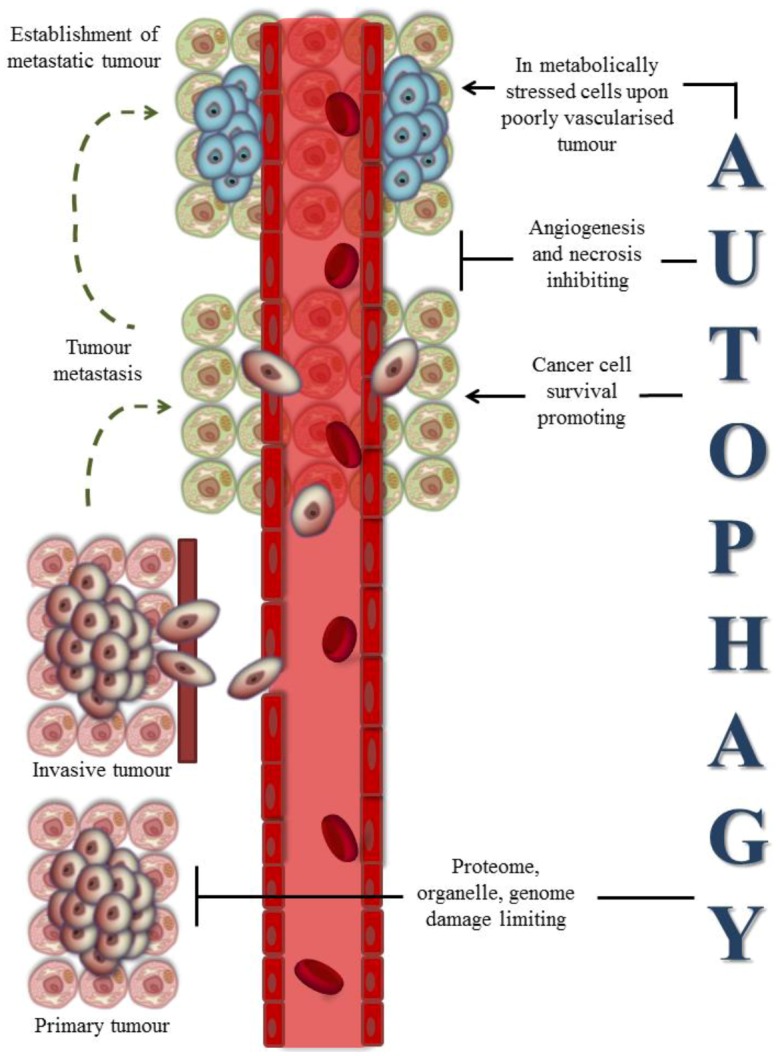
Multiple roles of autophagy during tumorigenesis. Autophagy can both inhibit and promote cancer formation through different mechanisms, depending on the stage of tumour.

A growing number of papers, reviewed in [74], suggest that numerous oncogenes (e.g., PI3K, activated Akt1 and antiapoptotic Bcl-2 family proteins) and oncosuppressor proteins (e.g., DAPK1, PTEN, TSC1 and TSC2, p53, Beclin 1 and UVRAG) regulate both autophagy and cancer, highlighting the strict connection between the two processes. However, the relationship between autophagy and cancer is strongly contradictory depending on the variety of cancer pathologies, stages of the disease and experimental approaches. On the other hand, autophagy plays also a very complex and contradictory role during the development and progression phases of cancer [75], since it can both inhibit and promote cancer formation through different mechanisms, as indicated in Figure 2.

One of these mechanisms can be found in the recent finding that epigenetic changes as well as their regulators, such as DNA methyltransferases (DNMTs), histone methyltransferases (HMTs), histone demethylases (HDMTs), histone acetyltransferases (HATs) and histone deacetylases (HDACs), play a pivotal role not only in development and differentiation, but also in pathogenesis. DNA methylation, the most studied epigenetic alteration in cancer, was the first epigenetic alteration to be connected to tumour development in addition to miRNAs that have been described to be involved in modulation of a wide range of biological processes, including apoptosis and autophagy, and in many human diseases, including cancer [76,77,78]. The possibility that the level of autophagy in cancer cells is dynamically influenced by epigenetic factors was sustained as a consequence of mutations in autophagy-related genes, including oncogenes and oncosuppressor genes. Many examples can be drawn from literature: the expression of the aplasia Ras homologue member 1 (ARH1) gene, an oncosuppressor regulating autophagy, is repressed in many cancers due to hypermethylation of its promoter [79]; miRNA-106a is overexpressed in NB4 acute myeloid leukemia (AML) cells [80]; miRNA-221/222 modulate autophagic cell death in MCF-7 cells [81]; miRNA-376b plays an oncogenic role in Hun-7 and MCF-7 cells by attenuating rapamycin-induced autophagy [82]; miRNA-183 overexpression inhibits autophagic cell death in medullary thyroid cancer (MTC) cells [83]; miRNA-30a decreases autophagic activity in rapamycin-treated T98G cells [84]; miRNA-101 is lost in several cancer types including breast, liver and prostate cancers [85].

It is worth noting that cancer cells can also mediate a bystander effect and induce autophagy in adjacent fibroblasts and other stromal cells to maintain homeostasis and growth of cancer cells [86], which strongly supports the role of autophagy in malignant cells’ self-preservation during chemo- and radiotherapy [87].

### 3.2. Autophagy and Cancer Therapies

Cancer treatments include traditional, *i.e*., surgery, radiation, and chemotherapy, and specialized, *i.e*., immunotherapy, hormone therapy, photodynamic therapy, radiotherapy and gene therapy, strategies. In its most general sense, the treatment of tumour by chemotherapy is based on chemicals efficient at killing cancerous cells, most likely by inducing apoptosis. However, accumulating evidences suggest a close relation between apoptosis and autophagy in terms of cell fate and involvement in cancer [88,89,90,91,92,93,94,95], that significantly impacts the curative effects of cancer therapies. Thus, autophagy could be considered a new target for cancer therapy since it is able to bypass the resistance of cancer cells to apoptosis during chemotherapy by inducing autophagy and autophagic cell death [96].

Particularly in chemotherapy, two major developments of therapeutic strategies in the clinical trials can be exploited: (1) to improve the killing efficacy of chemotherapy drugs or resensitize the chemoresistant cells to drugs by inhibition of the cytoprotective autophagy combined with anti-cancer drugs; (2) to drive the apoptosis-defective cancer cells towards autophagic cell death [97]. 

On the other hand, during chemotherapeutic strategies, induction of autophagy can occur to promote cell survival of cancer cells under the harsh conditions established in the disturbed microenvironment. In fact, autophagy is known to protect the cells against stresses (e.g., increased level of ROS), and damages (e.g., unfolded proteins, altered DNA and dysfunctional organelles) [96]. Thus, tumour cells’ resistance to drugs compromises the curative efficacy of chemotherapy. For example, autophagy elicits resistance to tamoxifen [98], anti-HER2 monoclonal antibody trastuzumab [99] and bortezomib [100] in breast cancer cells; moreover, autophagy occurrence delays apoptotic cell death in the human intestinal colon cancer cell line HT-29 treated with sulindac sulfide [101]. In this context, growingly efforts are carried out to combine chemotherapeutic agents and autophagy inhibitors. 

The most common autophagy inhibitors affect the formation of autophagosomes, *i.e.*, 3-methyladenine (3-MA) and wortmannin, the acidification of lysosomes, *i.e.*, chloroquine (CQ) and hydroxychloroquine (HCQ), and the fusion of autophagosomes with lysosomes, *i.e.*, bafilomicynA1 (BafA). These inhibitors are also used in experimental trials evaluating the efficacy of combined treatments with anti-cancer drugs as tyrosine kinase, histone deacetylase (HADC) and angiogenic inhibitors, antimetabolites and arsenic trioxide.

CQ or BafA combined with tyrosine kinase inhibitors, such as imatinib, nilotinib or dasatinib, increase cell death in chronic myeloid leukemia (CML) cells [102]; combination of imatinib with autophagy inhibition by using RNAi-mediated silencing of autophagy regulators (ATGs) or antimalarial lysosomotrophic agents represents a strategy to promote *in vitro* and *in vivo* cytotoxicity towards Gastrointestinal Stromal Tumors (GISTs) and to diminish both cellular quiescence and acquired resistance in GIST patients [103]. Moreover, inhibition of late stage of autophagyby BafA enhances imatinib-induced cytotoxicity in U87-MG and U373-MG glioma cells through mitochondrial disruption-induced apoptosis [104]. In addition, CQ combined with saracatinib increases mortality and slows down tumour growth rate in mice-bearing prostate cancer [105]. The presence of CQ during treatment of CML with HADC inhibitor suberoylanilide hydroxamic acid increases cell death in a p53 independent manner [106]. The anti-cancer effect of fluorouracil (5-FU) is improved by enhancing apoptosis in *in vitro* and *in vivo* colon cancer cells by co-treatment with 3-MA [107,108]. 3-MA combined with arsenic trioxide ignites apoptotic and autophagic cell death of leukemia cells [109]. 

In chemotherapy, not only autophagy inhibition but also autophagy induction could have a high therapeutic value since it could circumvent defective or blocked apoptosis in cancer cells [110]. The induction of autophagy occurs via mTOR or Bcl2 family protein inhibitors. Among mTOR inhibitors, rapamycin ensures cell growth inhibition and cell death induction in lymphoma cell lines and in malignant glioma, breast cancer, renal cell carcinoma and mesothelioma [111]. Bcl-2 inhibitors, such as BH3-mimetic gossypol, promote resensitization of U343 and U87 malignant glioma [112] and prostate cancer [113] cells to chemotherapeutic agents by induction of autophagic cell death.

Recently, the role of autophagy in antitumor immune cells, that play an important role in controlling cancer progression, has begun to be considered since conventional cancer therapies occasionally can interfere with the immune system [114]. Autophagy may be exploited to reprogram immune cells’ metabolism affected by anti-cancer drugs [115]. Wildenberg [116] demonstrated that 3-MA-mediated autophagy suppression hyperstabilizes the DCs-CD4+ T cells resulting in T-cell activation increase. Moreover, DCs take advantage of autophagy induction to promote and enhance cross-presentation of tumor antigens on Major Histocompatibility Complex (MHC) class I for cytotoxic T-lymphocyte (CTL) activation [117] and on MHC class II for T-helper (Th) cell activation [118]. Indeed, metabolism plays a very important role during differentiation of immune subsets, such as CD4+ T lymphocytes [119], and autophagy supports this phenomenon [120]. In addition, after T cell maturation and their displacement to the periphery, autophagy ensures survival by degradation of essential components of the apoptotic cell machinery [120] and maintenance of mitochondrial turnover [121,122]. Moreover, in bone marrow hematopoietic cells, induction of autophagy supports metabolism through the liberation of biosynthetic precursor, since activation of CD4+ T cells correlates with cytokine secretion, ATP production, fatty acid utilization and glycolytic activity reduction [123]. Thus, autophagy may exert a pivotal effect on overall patient survival during cancer therapies that negatively affect immune effectors.

In addition, cancer cells’ autophagy can also provide immunogenic tumor antigens, increase the efficiency of cross presentation and regulate antigen delivery via the release of autophagosomes [124,125]. By inducing autophagy and inhibiting proteasome and lysosome proteins degradation, Yi and coworkers [126] demonstrated that autophagosomes released by cancer cells contain a lot of ubiquitinated antigens that are very efficient in *in vitro* and *in vivo* CD8+ T cells cross-presentation and activation. Autophagosomes in cancer cells can also package Damage Associated Molecular Patterns (DAMPs), the effectors of immunogenic cell death, that can activate, upon release, the innate immune response and stimulate crosstalk between DCs and T cells [127]. These tumor-derived autophagosomes are considered an ideal vaccine candidate for cancer immunotherapy, as demonstrated in 3LL Lewis lung cancer and B16F10 melanoma models [128].

Autophagy induction is a common outcome also in cancer PDT and occurs in a variety of cell lines photosensitized with a broad spectrum of PSs. Autophagy, whose onset could be concomitant and/or precedent and/or consequent to PDT-induced apoptosis, contributes to death/survival balance in cancer PDT as recently reviewed by us [129]. Indeed, autophagy in PDT plays a pro-survival role in apoptosis competent cells and a pro-death role in deficient ones (as reviewed in Reiners *et al.* [130]). In PDT the role of autophagy depends on the primary site of organelles’ PS accumulation, ROS type, oxidative injury and molecular targets involved [131]. In particular, mitochondrial- and ER-localized PSs trigger a pro-survival autophagic response to recycle injured organelles [132,133], while, PSs localized and damaging lysosomes block autophagosome formation, thus leading to autophagy inhibition. It has been further demonstrated that also PSs localizing in mitochondria or ER after relocation from their primary damage site, induce autophagic cell death [134,135] in addition to apoptosis [136,137]. The kinetic of apoptosis/autophagy switch strictly depends on cell type, PS type and concentration and light dose. Mounting evidences suggest that PDT stimulates autophagy occurrence by photooxidation/inactivation of autophagic negative regulators, such as Bcl-2 and mTOR proteins [138,139] rather than key autophagic proteins, such as Beclin1, Atg5 and Atg7.

### 3.3. Nanomaterials-Induced Autophagy: A Tool in Cancer Therapy?

Recently, huge progress has been made in characterizing the autophagy process and its regulatory pathways, resulting in an explosion of autophagy applied research. The impact of NMs on autophagic processes takes place in this context. In fact, a growing body of literature suggests that intracellular NMs may be selectively compartmentalized upon autophagic sequestration. In fact, NMs have been observed within autophagosomes present in alveolar macrophages, non-small cell lung cancer cells, human mesenchymal stem cells, dendritic cells, and murine macrophages and human lung adenocarcinoma treated with carbon black NPs, EGFR-targeted gold-coated iron oxide NPs, quantum dots, alumina NPs and silica NPs, respectively [140,141,142,143,144]. However, several reports recently reviewed in [58], also suggest that biopersistent NMs can, in turn, perturb autophagic pathways via induction and blockade in a wide variety of biological models. Nanomaterials can alter autophagy signaling pathways via (1) induction of oxidative stress-dependent signaling (e.g., ER stress, mitochondrial damage) [145,146,147], (2) suppression of Akt-mTOR signaling [148,149,150], and (3) alteration of autophagy related gene/protein expression [151]. 

**Figure 3:**
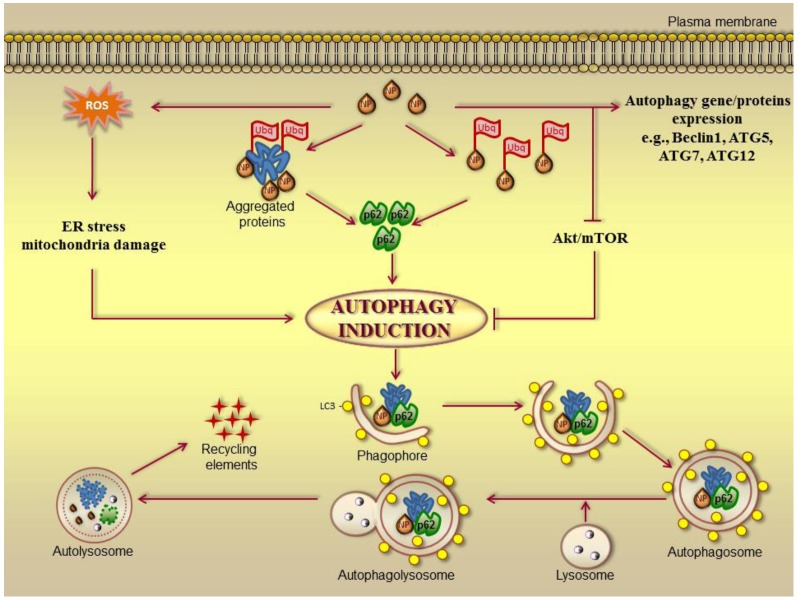
Nanoparticle-induced autophagy (adapted from [58]).

Cells may select nanomaterials for autophagy through a p62-LC3 II pathway upon ubiquitination of nanomaterials directly or indirectly through colocalization with protein aggregates in a manner similar to invading pathogens one [152]. A hypothesized mechanism involved in autophagy perturbation induced by NMs is reported in Figure 3.

Consistent with *in vitro* findings, autophagic hallmarks were also observed in *in vivo* models. In fact, autophagosomes accumulation was detected in the lung tissue of mice treated with cationic dendrimers and carboxylated carbon nanotubes [153].

Finally, since NMs interact with autophagy pathway, they are also proposed as tools exploiting in autophagy monitoring [154]. The NMs-derived autophagy dysfunction is not necessarily always disadvantageous since it is considered a cancer therapeutic mechanism in many novel nanomedicine therapeutics. In fact, due to autophagic pro-survival and pro-death dual role, both autophagy blockade and induction elicited by NMs could be exploited in terms of cancer therapies. In Table 2 we report a summary of NPs types modulating the autophagic process in cancer treatment.

In cancer treatment, NPs can be used alone or in combination with different types of molecules, such as chemotherapeutics, antigen or antibodies, in order to enhance susceptibility of cancer cells to death through modulation of autophagy.

**Table 2:** A summary of NPs-mediated autophagy in cancer treatment.

Nanomaterials	Model	Delivered molecule	Reference
GNP-Chl	MCF-7 human breast cancer cells	Chloroquine	[155]
Fe@Au-NPs	OEMC1 human oral cancer cells	-	[146]
Magnetic NPs (C225-NPs)	NSCLC non-small cell lung cancer cells	Anti-EGFR antibody	[144]
C_60_(Nd)-NPs	HeLa cervix cancer cells MCF-7 cells	doxorubicin	[156]
nC_60_	C6 rat glioma cells U251 human glioma cells	-	[145]
FeO-NPs	A549 human lung epithelial cancer cells	-	[147]
nC_60_	HeLa cervix cancer cells MCF-7 cells	Doxorubicin	[151]
α-Al_2_O_3_-NPs	C57BL/6 with 3LL lung tumour	Antigen tumour derived	[141]

Wu and coworkers [146] suggest that NPs with an iron core and gold shell (denoted Fe@Au) limit cancer cell proliferation as already demonstrated for magnetic NPs [157]. In particular, Fe@Au-NPs induce a cancer-specific cytotoxicity via mitochondria-mediated autophagy in OECM1 oral cancer cells. Fe@Au-NPs cause an irreversible mitochondrial potential loss and ROS production only in cancer cells and not in healthy ones.

Magnetic NPs (C225-NPs), consisting of a paramagnetic iron core surrounded by a gold layer, were used to target anti-EGFR antibody in order to improve cell killing of human non-small cell lung cancer (NSCLC) cells [144]. The epidermal growth factor receptor (EGFR) is overexpressed in 80% of NSCLC [158] and EGFR-targeted inhibitors are used in the clinic for NSCLC [159,160,161], but the survival rate of the patients is less than 16%. The nanoscale three-dimensional arrangement of anti-EGFR antibody by using NPs provides a tool to increase efficacy of therapy against lung cancer cells. In fact, C225-NPs treatment regulates EGFR-signaling pathway and produces greater antitumour activity by apoptotic and autophagic cell death in lung tumor cells than in normal cells. The density of anti-EGFR antibody attached to NPs is crucial in C225-NPs-mediated tumour cell killing [144]. 

A cytotoxic action towards cancer, but not normal cells, is displayed by bare magnetic NPs, like iron oxide (FeO)-NPs, via ROS production and mitochondrial membrane alteration-mediated autophagy [147]. In fact, FeO-NPs induce cell death and ROS generation, affecting mitochondrial membrane potential, in human lung epithelial cancer cells A549. ROS generation hyper-activates cell death via autophagy, as confirmed by the use of 3-MA autophagic inhibitor. Pre-treatment of A549 cells with Compound C (a selective and ATP-competitive inhibitor of AMPK) demonstrates that FeO-NPs induce autophagy through Akt-AMPK-mTOR pathway in cancer cells. Moreover, FeO-NPs can be considered a tool to selectively kill cancer cells since normal human lung fibroblast cells IMR-90 are unaffected by NPs treatment [147]. 

Due to its geometrical structure and unique physical and chemical properties, fullerene C_60_ is a potent anti-cancer engineered NM [162,163]. Even water-soluble C_60_ derivatives display cytoprotective or cytotoxic effects exploitable in cancer treatment. Particularly, nanocrystalline fullerene (nano-C_60_, nC_60_) displays *in vitro* cytotoxic action against glioma, incurable tumours notoriously resistant to chemotherapy [164] through multiple (oxidative stress-dependent and independent) mechanisms [145]. Depending on high (1 µg/mL) or low (0.25 µg/mL) dose, nC_60_ can cause necrosis or block proliferation in rat glioma cell line C6 and in human glioma cell line U251 respectively. High doses of nC_60_ induce ROS production, which in turn elicits necrosis, directly or via ERK activation. On the other hand, block of proliferation (low dose of nC_60_) depends on G2/M cell cycle arrest mediated by autophagy induction, as demonstrated by use of the BafA inhibition of autophagy. The fact that primary astrocytes are less sensitive to cytostatic action of nC_60_, suggests a possible tumour-specific targeting [145]. 

Non-cytotoxic concentrations of nC_60_ sensitize HeLa cells and drug-resistant MCF-7 cells to doxorubicin (Dox)-mediated death. In addition, nC_60_ sensitizes HeLa cells to cisplatin-mediated death. The enhanced nC_60_ chemosensitizationis autophagy-mediated and requires a functional ATG5, a key gene in autophagy signaling pathway [151]. 

A greater induction of autophagy and sensitizing potential to low dose of Dox in an autophagy-dependent manner, has been reported for a novel nC_60_ derivative, nC_60_(Nd) in HeLa and MCF-7 cells [156]. It is likely that, autophagic pathway is blocked after autophagosome/lysosome fusion stepand nC_60_(Nd) was not degraded due to its inorganic nature [156]. 

Alpha-alumina NPs (α-Al_2_O_3_-NPs) are exploited to design a novel therapeutic vaccine by induction of autophagy; in fact, AlNPs can be used to transport bound antigen to autophagosomes in dendritic cells (DCs) in an p62-mediated manner eliciting a potent T-cell anti-tumor response [141]. Therapeutic cancer vaccination is an attractive strategy in oncology to actively induce T cells to specifically recognize and destroy ill cells in cancer patients. The use of NMs in cancer vaccination could overcome the lack of a large number antigen-specific T cells that are not ensured in conventional vaccine carrier systems [165] or can reduce the amount of antigen required by DCs to activate T cells *in vivo* as for α-Al_2_O_3_-NPs as antigen carrier. In fact, DCs pulsed with α-Al_2_O_3_-NPs conjugated with autophagosomes are able to boost T cell response that suppresses the formation of metastases in C57BL/6 mice bearing experimental metastases 3LL lung tumours [141]. Finally, NPs can elicit cancer cell death *in vitro* via autophagy induction in combination with a drugs such as CQ, that is not a chemotherapeutic molecule *per se*, but an excellent chemosensitizer under investigation in combinatorial therapy [166]. In particular, Joshi *et al*. [155] demonstrated that CQ-conjugated gold NPs (GNP-Chl) exhibit concentration-dependent cytotoxicity in MCF-7 cells by triggering autophagy-mediated necrotic cell death.

## 4. Conclusions

The exploitation of the autophagy pathway as a new cancer therapeutic option is under investigation. Autophagy plays a complex role in cancer cells depending on the phases of carcinogenesis and tumour context. Literature data demonstrate that both autophagy enhancers and inhibitors may provide beneficial or adverse effects for cancer treatment Defective autophagy can allow genomic instability leading to tumor onset and development as well as up-regulated autophagy in growing tumors can fight nutrient deprivation. On the other hand, up-regulation of autophagy can also represent a tool to induce cell death in cancer cells. However, the concomitant use of autophagy inhibitors and anti-neoplastic drugs improve chemotherapy efficacy. In this context, the continued progress of nanotechnology could promote very important amelioration for both the diagnosis and treatment, and not only In fact, it has recently been demonstrated that engineered NMs can impact positively and negatively on the autophagic pathway, and once properly engineered can recognize disease at the cellular level, be visible on imaging studies, and deliver therapeutic compounds. Thus, on the one hand, nanotechnology could enable earlier detection and treatment of diseases that are best treated in their initial stages. On the other hand, advances in nanotechnology will spur the discovery of new methods for delivery of therapeutic compounds, including genes and proteins, to diseased tissue. Thus, understanding the synergy between autophagy, cancer therapy and NMs could be pivotal to design new therapeutic cancer strategies, in consideration also of the recent findings linking NMs to long-term impact on genetic and epigenetic processes and to autophagic process regulation by miRNAs. 
